# Mussel oil is superior to fish oil in preventing atherosclerosis of ApoE^−/−^ mice

**DOI:** 10.3389/fnut.2024.1326421

**Published:** 2024-02-12

**Authors:** Kelei Li, Xiaolei Song, Huiying Li, Xiaotong Kuang, Shiyi Liu, Run Liu, Duo Li

**Affiliations:** ^1^Institute of Nutrition and Health, Qingdao University, Qingdao, China; ^2^School of Public Health, Qingdao University, Qingdao, China

**Keywords:** atherosclerosis, mussel, lipids, inflammation, smooth muscle cell, NF-κB, p38MAPK, VCAM-1

## Abstract

**Objectives:**

The present study aimed to explore the preventive effect of mussel oil (MO) on atherosclerosis and the potential mechanism in apolipoprotein E-null (ApoE^−/−^) mice.

**Methods:**

ApoE^−/−^ mice were fed with a high-fat and high-cholesterol chow and given corn oil (CO), fish oil (FO), MO, or aspirin (ASP, dissolved in CO) by gavage for 12  weeks. The total n-3 polyunsaturated fatty acids (PUFAs) in MO (51.01%) and FO (46.82%) were comparable (mainly C22:6n-3 and C20:5n-3). Wild-type mice were fed with a normal chow and given equivalent CO as health control (CON).

**Results:**

Compared with the CON group, obvious atherosclerotic plaque appeared at aorta and aortic sinus in the CO group. Compared with the CO group, MO but not FO had a significantly smaller atherosclerotic plaque area in the aorta. The aortic atherosclerotic plaque area was comparable in the MO, CON, and ASP groups. The MO group had a significantly smaller atherosclerotic plaque area, lower lipid deposition, lower contents of smooth muscle cell (SMC), and slightly lower contents of macrophage at the aortic sinus than the FO group. Serum concentrations of IL-1β, NF-κB, and VCAM-1 were comparable in the MO and FO groups and were significantly lower than the CO group. Compared with the CO group, the MO group but not FO group had significantly lower aortic protein levels of p65NF-κB, p38MAPK, and VCAM-1. The aortic protein levels of p-p65NF-κB and p-p38MAPK were significantly lower in the MO group than the FO group.

**Conclusion:**

In conclusion, MO is more potent than FO in preventing atherosclerosis, and the possible mechanism may be by downregulating p38MAPK/NF-κB signaling pathway, decreasing VCAM-1 and macrophage, and inhibiting proliferation and migration of SMC.

## 1 Introduction

Atherosclerosis is one of the most important causes of coronary artery disease, carotid artery disease, and peripheral arterial disease (1). Dysregulation of lipid metabolism and chronic inflammation are key triggers of atherosclerosis (2, 3).

In recent years, the beneficial effect of functional lipids on atherosclerosis has been paid much attention, and one of the most representatives is n-3 polyunsaturated fatty acid (PUFA)-enriched oil, such as fish oil (FO). In low density lipoprotein (LDL) receptor knock-out mice, FO supplementation led to a significantly lower atherosclerotic lesion area (4). Dietary intake of C20:5n-3 decreased the area of atherosclerosis lesions in apolipoprotein E-null (ApoE^−/−^) mice (5), one of the most widely used atherosclerosis models with lesions comparable to human lesions (6). A prospective cohort study observed a negative association between n-3 PUFA intake and the risk of carotid intima-media thickness (7). Another cohort study found a negative association between plasma C20:5n-3 and risk of cardiovascular disease events, and this association is more apparent in subjects with a higher score of coronary artery calcium (8). Potential mechanism is conducted by anti-inflammation and improvement in lipid metabolism (9–11).

Mussel oil (MO) contains a high content of n-3 PUFA (mainly C20:5n-3 and C22:6n-3) (12). Our latest studies found that MO had a beneficial effect on glycemic traits in both humans and mice and was superior to FO having comparable content of total n-3 PUFA (12, 13). It is noteworthy that MO also has a terrific anti-inflammatory effect. Our previous randomized controlled trials (RCTs) observed that MO improved clinical conditions of patients with rheumatoid arthritis and decreased serum levels of pro-inflammatory cytokines and eicosanoids (14). A better anti-inflammatory effect of MO than FO was observed in patients with type 2 diabetes mellitus (T2DM) (13). Another study extracted furan fatty acids from MO and found that they had a much better anti-inflammatory effect than C20:5n-3 in adjuvant-induced arthritis rats (15). In addition, MO also has a better lowering effect than FO on serum triacylglycerol (TG) (13).

Considering the beneficial role of MO in inflammation and lipid metabolism, we speculate that MO may have an anti-atherosclerosis effect, but this has not been verified in previous studies. Therefore, the aim of the present study was to explore the effect of MO on atherosclerosis and the potential mechanism by using ApoE^−/−^ mice.

## 2 Materials and methods

### 2.1 Ethical approval

The study was approved by the Ethics Committee of Medical College of Qingdao University (QDU-AEC-2022369). All animal experimental procedures were performed in accordance with the Guidelines for Care and Use of Laboratory Animals of Qingdao University.

### 2.2 Treatment oils

Mussel meat was vacuum freeze-dried and homogenized into powder, and then, MO was separated by supercritical fluid extraction (China Harbin Essen Biotechnology Co., Ltd.) (16). FO was purchased from Longzhou Biotechnology Co., Ltd., Xi’an, China. Corn oil (CO) was purchased from a local supermarket (Brand: LONGEVITY FLOWER).

### 2.3 Study design

**Figure 1:**
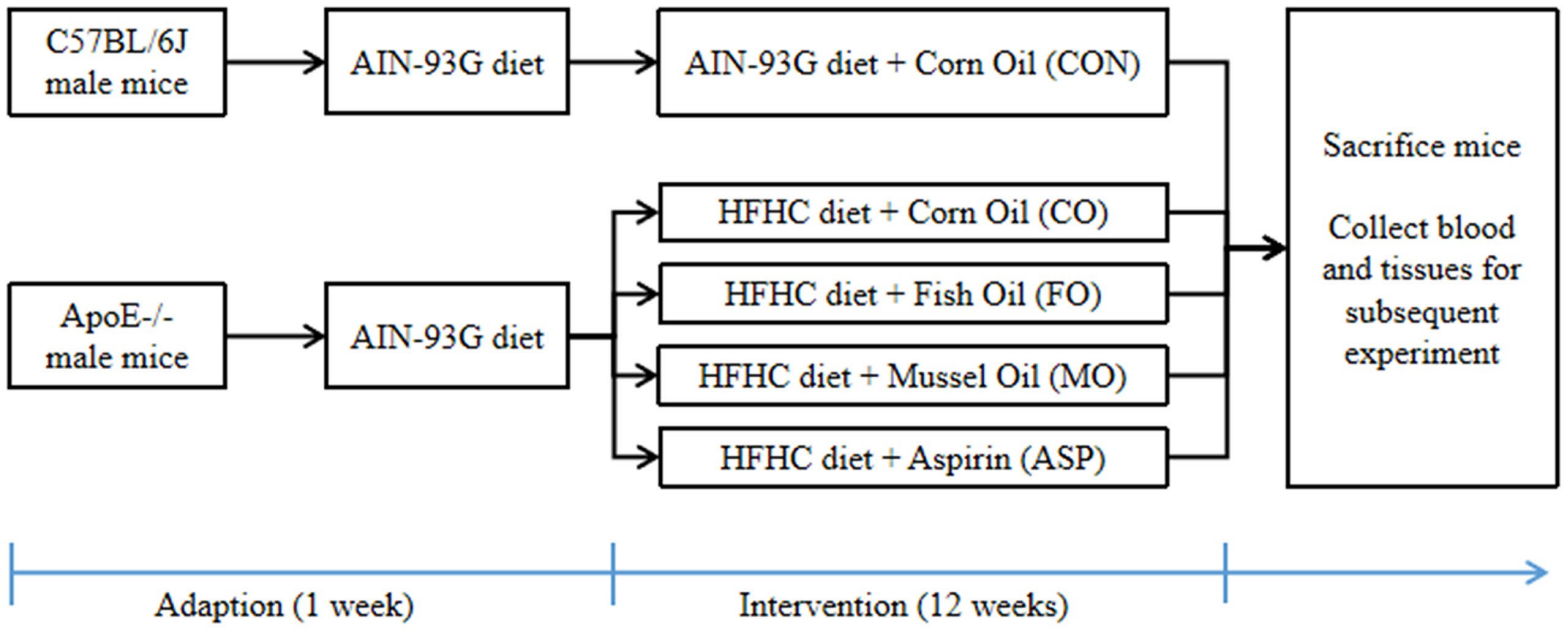
Diagram of study design. HFHC, high-fat and high-cholesterol. Aspirin was dissolved in corn oil (0.5 mg/mL). The dosage of each oil was 0.1 mL/10 g/day.

The study design is shown in Figure 1. In brief, 6-week-old male wild-type C57BL/6 J mice (*n* = 6) and ApoE^−/−^ C57BL/6 J mice (*n* = 24; Beijing Vital River Laboratory Animal Technology Co., Ltd) were housed in standard laboratory cages in a specific pathogen-free room under standard conditions (21 ± 1°C, 60% humidity, and 12 h light/dark cycle). After 1 week of adaption, ApoE^−/−^ mice were randomly divided into four groups and fed with a high-fat and high-cholesterol (HFHC) chow (21% fat and 0.15% cholesterol, w/w), given CO, FO, MO, or aspirin (ASP, 0.5 mg/mL, and dissolved in CO) by gavage. The dosage of each oil is 0.1 mL/10 g/day. Wild-type mice were fed with a normal chow (AIN-93G) and given equivalent CO by gavage as health control (CON). The detailed fatty acid compositions of treatment oils are shown in Supplementary Table S1. In brief, the total n-3 PUFA content in MO and FO was 51.01 and 46.82%; MO had a slightly higher C22:6n-3 (26.72% vs. 19.34%) and a slightly lower C20:5n-3 (20.66% vs. 25.27%) than FO; the content of C18:2n-6 in CO was 53.47%. After 12 weeks of treatment, mice were sacrificed to collect tissues and blood samples for subsequent detection.

### 2.4 Atherosclerotic plaque quantitation and histologic analysis

To detect atherosclerotic plaque in the aorta, the entire aorta was put in PBS solution (0.01 M, pH 7.2–7.4), carefully stripped of perivascular fat under stereoscopic microscope, fixed with 4% paraformaldehyde, and cut open longitudinally and stained by Oil-red O (17, 18). Serial cryosections were obtained at the aortic sinus and aortic arch for histological analysis. In brief, the tissues were embedded, and serial 8–10 μm thick sections were cut from the aortic root for observation under microscope. Once three valves were observed, the sections were retained for staining. The plaque and lipid deposition of aortic arch and aortic sinus was quantified by staining sections with hematoxylin–eosin (H&E) and Oil-red O, respectively. The macrophage and the smooth muscle cell (SMC) of aortic sinus were detected by immunohistochemically staining with CD68 and anti-a-smooth muscle actin (a-SMA) antibodies, respectively (17, 19). Collagen of aortic sinus was detected by Sirius red staining (17). IPWIN32 software was used to quantify the plaque area, lipid deposition, macrophage, SMC, and collagen. The atherosclerotic lesion area in the aorta en face was quantified as a percent of the aortic surface area (20). The outline of the atherosclerotic lesions in aortic sinus and aortic arch was marked with a black dashed line. The positive regions of CD68 and α-SMA are brownish yellow, and the positive regions of Oil-red O and Sirius red staining are red. Lipid deposition in the aortic sinus and aortic arch and CD68, α-SMA, and Sirius red staining in the aortic sinus were expressed as a ratio of positive area versus atherosclerotic plaque area (21).

### 2.5 Fatty acid determination

We analyzed erythrocyte membrane phospholipid (PL) fatty acid composition, which can reflect the changes in response to long-term dietary fat intake (22, 23). The fatty acid compositions (% in total fatty acids) in the treatment oil were detected according to our previous study (12). Moreover, the erythrocyte membrane was separated and washed according to our another previous study (24). In brief, lipids of erythrocyte membrane were extracted by chloroform/methanol (1:1), and the phospholipid (PL) fraction was separated by thin-layer chromatography. The lipids within the treatment oils and the PL fraction of erythrocyte membrane were blended with toluene and 0.9 mol/L H_2_SO_4_/methanol (1:3, v:v). Fatty acid methyl esters were generated by incubating the mixture at 70°C for 120 min. They were extracted using n-hexane and then purified using a Sep-Pak silica column. Subsequently, the solution was dried under N_2_ and redissolved in n-hexane. Fatty acid methyl esters of the treatment oil and erythrocyte membrane PL were separated by a gas chromatography (GC) equipped with an Agilent DB-23 column (60 m, 0.25 mm*0.25 μm). The sample inlet temperature was maintained at 260°C, while the pressure of N_2_ and H_2_ was set to 50 and 75 kPa, respectively. The temperature program of the GC was as follows: 0–2 min at 140°C; 2–3 min ramping to 160°C; 3–8 min at 160°C; 8–9 min ramping to 180°C; 9–21 min at 180°C; 21–22 min ramping to 200°C; 22–30 min at 200°C; 30–30.25 min ramping to 205°C; and 30.25–41.25 min at 205°C. A standard of fatty acid mixture (cat. no. 18919-1AMP SUPELCO, Sigma–Aldrich) was used to identify individual fatty acids according to retention time.

### 2.6 Determination of serum lipids and inflammatory factors

Serum lipids were detected by biochemical kits (Nanjing Jiancheng Bioengineering Institute): TG (A110-1-1), total cholesterol (TC; A111-1-1), high density lipoprotein cholesterol (HDL-C; A112-1-1), and low density lipoprotein cholesterol (LDL-C; A113-1-1). Serum inflammatory factors were detected by ELISA kits (Shanghai Jining Industrial Co, Ltd): interleukin-6 (IL-6; JN16894), IL-10 (JN17305), IL-1β (JN16939), vascular cellular adhesion molecule-1 (VCAM-1; HN20565), nuclear factor kappa-B (NF-κB; JN20529), tumor necrosis factor-α (TNF-α; JN17113), and Monocyte Chemoattractant Protein-1 (MCP-1; JN17005).

### 2.7 Determination of protein levels in the aorta

Protein levels of genes in the aorta were detected by Western blotting analysis. In brief, aortic tissue lysates were separated by 10% SDS-PAGE and transferred to 0.45 μm PVDF membranes (IPVH00010, Merck Millipore). After blocking in 5% skim milk (Cat#D8340, Solarbio; for non-phosphorylated protein) or 5% BSA (G5001-5G, Servicebio; for phosphorylated protein) for 2 h, the membranes were incubated overnight at 4°C with primary antibodies: p65NF-κB (1:1000, AF5006, Affinity Biosciences); p-p65NF-κB (1:1000, AB76302, Abcam); p38 mitogen-activated protein kinase (p38MAPK; 1:2500, ab170099, Abcam); p-p38MAPK (1:1000, AB195049, Abcam); VCAM-1 (1:5000, ab134047, Abcam); and β-actin (1:1000, GB15003, Servicebio). The membranes were then incubated with HRP-conjugated secondary antibody for 90 min at room temperature. Protein bands were visualized using ECL solution on Ultra Sensitive Multifunctional Imager (AI680RGB, GE, Japan) and analyzed using the ImageJ software.

### 2.8 Determination of furan fatty acids and astaxanthin

The contents of 11-(3,4-dimethyl-5-propylfuran-2-yl)undecanoic acid (11D3) and 11-(3,4-dimethyl-5-pentylfuran-2-yl)undecanoic acid (11D5) were quantified by Agilent Technologies 6530C Q-TOF UPLC-MS/MS, according to previous studies (25, 26). In brief, serum (20 μL), internal standard (100 ng), and 5% KOH-ethanol (500 μL) were added to a centrifuge tube and kept at 60°C for 2 h. Then, the pH was adjusted to 4 with 1 M HCl. The reaction products were extracted with n-hexane (300 μL) three times. After evaporation of the solvent, 11D3 and 11D5 were derived into 11D3-3-acyl-oxymethyl-1-methylpyridinium iodide (AMMP) and 11D5-AMMP using 20 μLBMP (2-bromo-1-methyliodopyridine, 7.5 mg/mL in acetonitrile), 20 μl CMP (3-methanol-1-methyliodopyridine, 10 mg/mL in acetonitrile), and 1 μL triethylamine. After derivatization, the solution was dried under N_2_ again and redissolved in 100 μL of acetonitrile/H_2_O (7:3, v/v). The derivatized furan fatty acids were separated by UPLC equipped with a Sepax Opalshell C18 column (2.1 mm x 100 mm, i.d.2.6 μm) in positive mode. The mobile phase consisted of H_2_O (0.1%HCOOH; A) and acetonitrile (0.1%HCOOH; B). The gradient used was (min/% B): 0:10; 1:20; 4:30; 7:40; 13:50; 20:50; 21:60; 25:60; 26:70; 27:70; 28:100; 32:100; and 33:10. A flow rate of 0.4 mL min^−1^ was used, and the injection volume was 2 μL. The main fragmentations were: 11D3-AMMP (m/z 428–107, 428–124, 428–178) and 11D5-AMMP (m/z 456–107, 456–124, 456–178). The MS scan range was set at m/z 100–600 and the MS/MS analysis at collision energy of 42 V.

Astaxanthin content was quantified by high-performance liquid chromatography (HPLC) (26, 27). A certain amount of the sample was dissolved in 1 mL dichloromethane: methanol (1:3, v:v), saponified, and transferred into a capped test tube. The samples were determined by HPLC equipped with a C30 column (250 mm x 4.6 mm, 5 μm) at 25°C. The mobile phase consisted of methanol (A), tert-butyl methyl ether (B), and 1% phosphoric acid solution (C). The gradient used was (min/% B): 0:15; 15:30; 23:80; 27:80; 30:15; and 35:15. The mobile phase C remained at 4% throughout the process. A flow rate of 1.0 mL min^−1^ was used, and the column was monitored at 474 nm.

### 2.9 Statistical analysis

Data were expressed as mean ± SEM unless otherwise specified. One-way ANOVA was used for significance test, and an LSD post-hoc test was used for multiple comparisons between groups. Spearman correlation analysis was used to evaluate the linear relationship between continuous variables. Partial correlation was used to analyze the relationship between serum furan fatty acids and atherosclerosis-related parameters. A value of *p* < 0.05 was considered to be statistically significant. All statistical analyses were conducted using SPSS 26.0. Figures were generated using GraphPad Prism 8.02.

## 3 Results

### 3.1 Effect of mussel oil on atherosclerotic lesion formation

**Figure 2:**
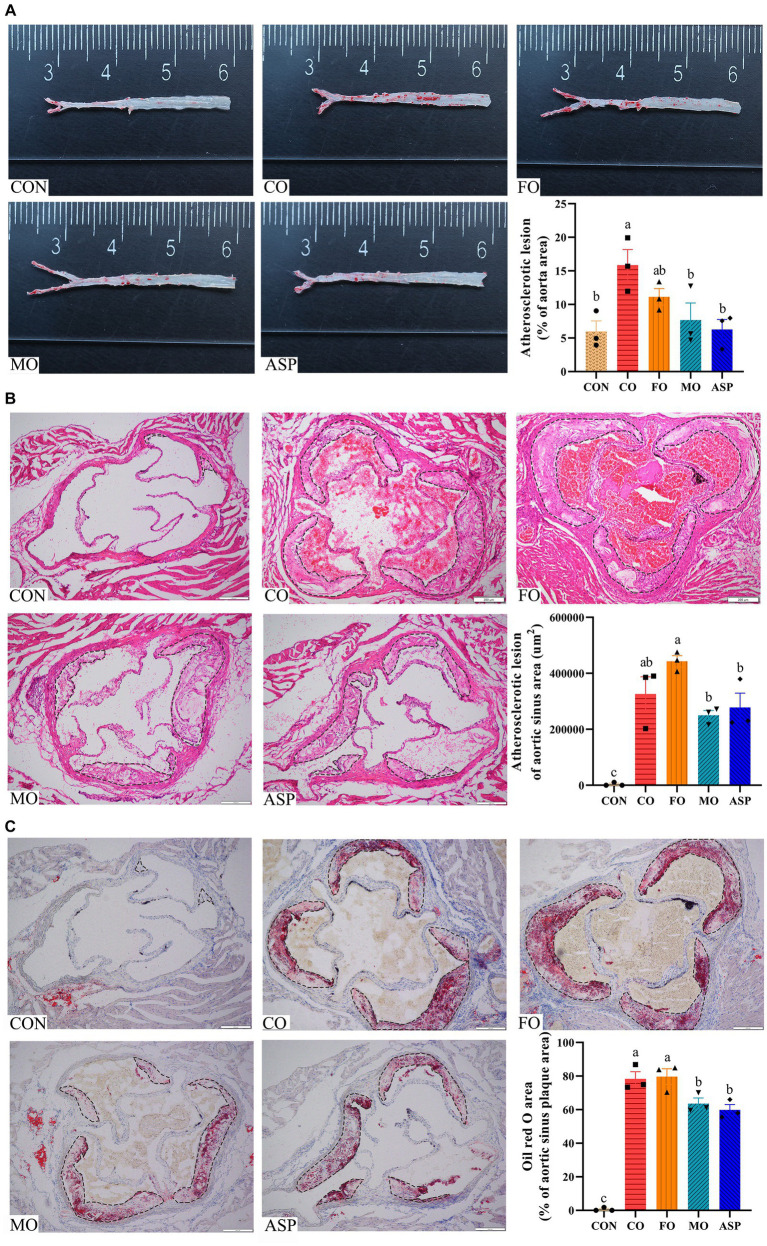
Effect of treatment oils on atherosclerotic plaque in the aorta **(A)** and aortic sinus **(B,C)**. Three mice in each group were included for analysis. For B–C, 2 serial sections of each mice were used, and the mean of two sections from one mice was included in the final analysis. Atherosclerotic plaque in the aorta was detected by Oil-red O staining **(A)**. Atherosclerotic plaque and lipid deposition in the aortic sinus was detected by H&E **(B)** and Oil-red O staining **(C)**, respectively. The outline of the atherosclerotic lesions in the aortic sinus was marked with a black dashed line. The result of Oil red O staining in the aortic sinus was normalized by atherosclerotic plaque area. Data were expressed as mean ± SEM. There was significance if groups did not share the same letter (*p* < 0.05). CON, health control; CO, corn oil; FO, fish oil; MO, mussel oil; ASP, aspirin.

**Figure 3:**
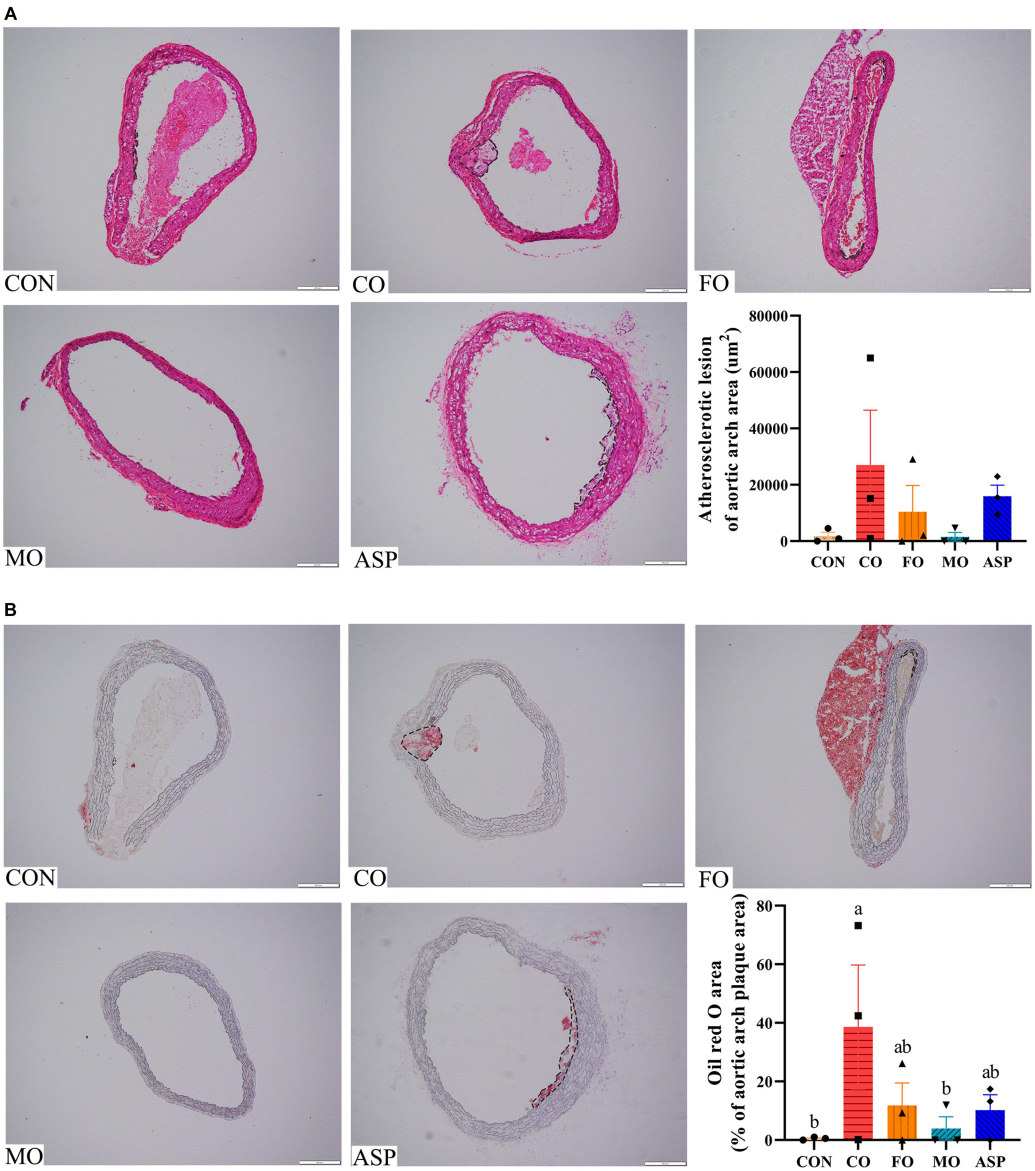
Effect of treatment oils on atherosclerotic plaque in the aortic arch. Three mice in each group were included for analysis, and two serial sections of each mice were used. The mean of two serial sections of each mice were used for analysis. **(A)** Results of H&E staining. **(B)** Results of Oil-red O staining. The outline of the atherosclerotic lesions in the aortic arch was marked with a black dashed line. The result of Oil red O staining was normalized by atherosclerotic plaque area. Data were expressed as mean ± SEM. There was significance if groups did not share the same letter (*p* < 0.05). CON, health control; CO, corn oil; FO, fish oil; MO, mussel oil; ASP, aspirin.

Compared with the CON group, significantly greater atherosclerotic plaque area in the aorta and aortic sinus was observed in the CO group (*p* < 0.05; Figure 2). The MO group had a significantly smaller atherosclerotic lesion area of the aorta than the CO group (*p* < 0.05), and it was comparable with that in the ASP and CON groups (*p* > 0.05). The atherosclerotic lesion area in the aorta of the FO group was slightly smaller than the CO group, but this difference was non-significant (*p* > 0.05). The atherosclerotic plaque area and lipid deposition of the aortic sinus were significantly lower in the MO group than in the FO group (*p* < 0.05). There was no significant difference in atherosclerotic lesion area of the aortic arch in the CON, CO, FO, MO, and ASP groups (*p* > 0.05; Figure 3). Significantly higher lipid deposition of the aortic arch was observed in the CO group than in the CON and MO groups (*p* < 0.05). The lipid deposition of the aortic arch in the MO group was slightly lower than in the FO and ASP groups, but this difference was non-significant (*p* > 0.05).

**Figure 4:**
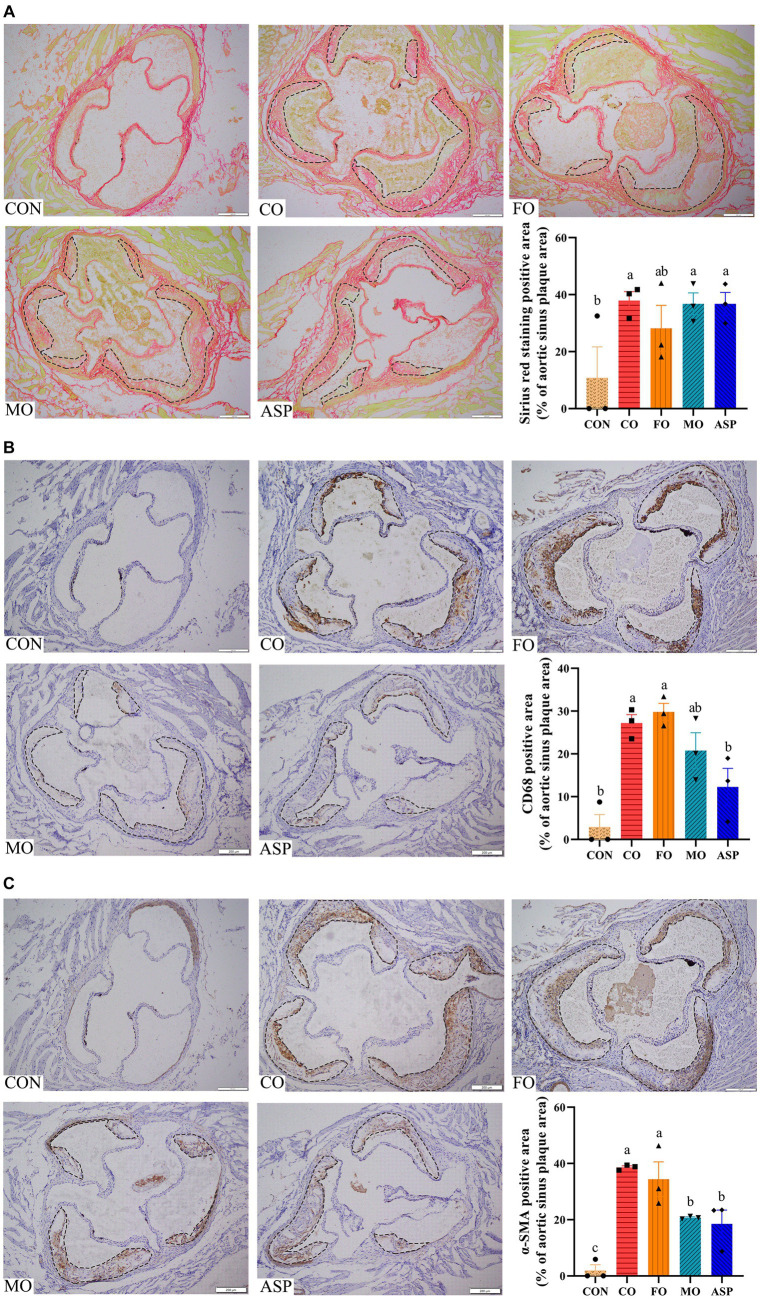
Effect of treatment oils on collagen **(A)**, macrophage **(B)**, and SMC **(C)** in the aortic sinus. Three mice in each group were included for analysis, and two serial sections of each mice were used. The mean of two serial sections of each mice were used for analysis. The collagen was detected by Sirius red staining. The macrophage and SMC were detected by immunohistochemically staining with CD68 and anti-a-smooth muscle actin (a-SMA) antibody, respectively. The positive areas were quantified within the range of plaques circled by the black dashed line. The results were normalized by atherosclerotic plaque area. Data were expressed as mean ± SEM. There was significance if groups did not share the same letter (*p* < 0.05). SMC, smooth muscle cell; CON, health control; CO, corn oil; FO, fish oil; MO, mussel oil; ASP, aspirin.

There was significantly fewer SMC (indicated by α-SMA) in the aortic sinus in the MO and ASP groups than in the CO and FO groups (*p* < 0.05; Figure 4). The CO, MO, and ASP groups had a significantly higher content of collagen (indicated by Sirius red staining) in the aortic sinus than in the CON group (*p* < 0.05). No significant difference in collagen was observed between the CO, FO, MO, and ASP groups (*p* > 0.05). The content of macrophages (indicated by CD68) in the aortic sinus of the CO and FO groups was significantly higher than in the CON group (*p* < 0.05). The ASP group had significantly lower content of macrophages than in the FO group (*p* < 0.05), and the MO group had slightly lower content of macrophages than in the FO group (*p* = 0.077).

### 3.2 Effect of mussel oil on serum lipids and inflammatory factors

**Figure 5:**
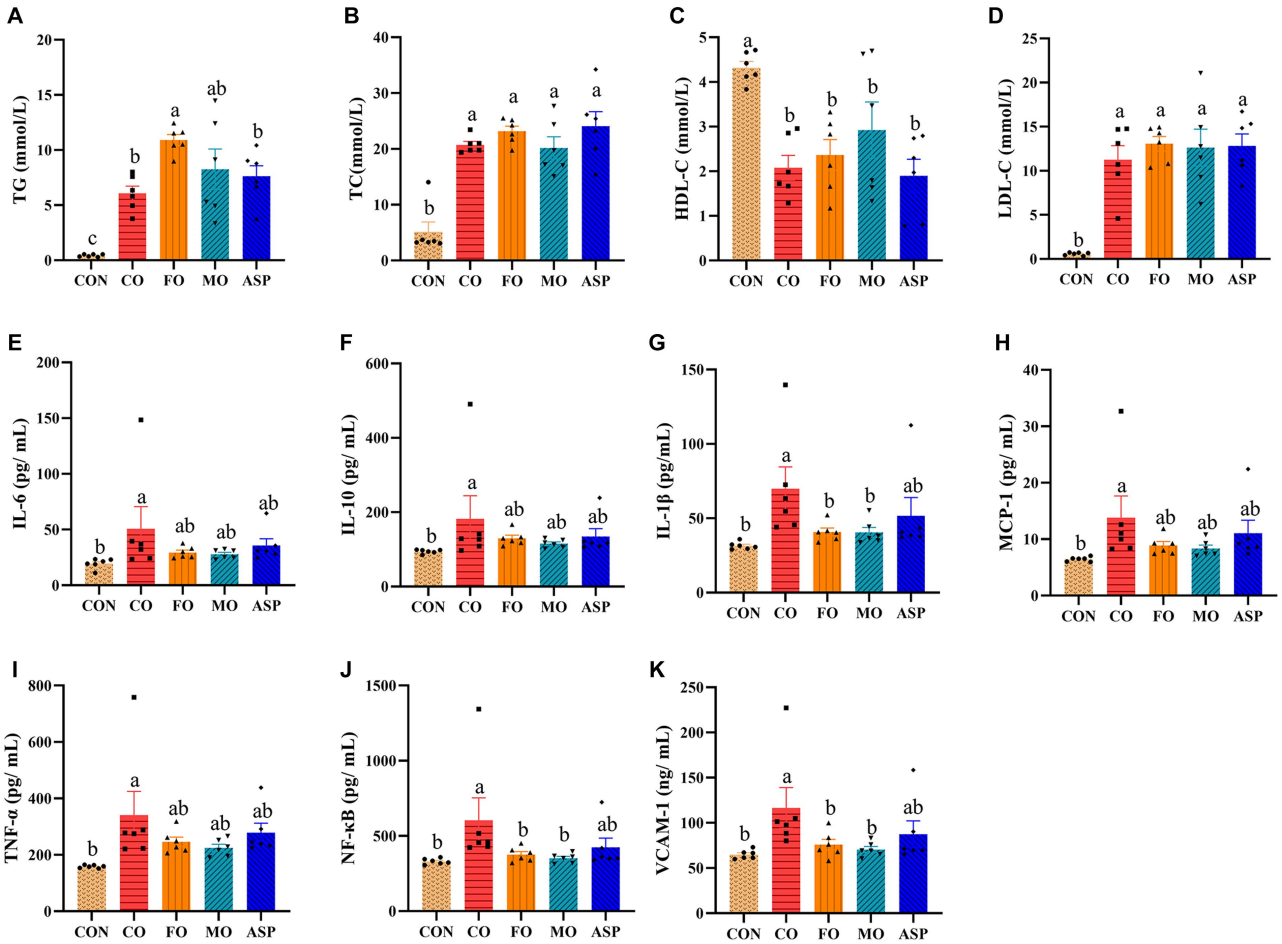
Effect of treatment oils on serum lipids and inflammatory factors (*n * =  6 in each group). The results of TG **(A)**, TC **(B)**, HDL-C **(C)**, LDL-C **(D)**, IL-6 **(E)**, IL-10 **(F)**, IL-1β **(G)**, MCP-1 **(H)**, TNF-β **(I)**, NF-βB **(J)** and VCAM-1 **(K)**. Data were expressed as mean  ±  SEM. There was significance if groups did not share the same letter (*p*  <  0.05). CON, health control; CO, corn oil; FO, fish oil; MO, mussel oil; ASP, aspirin.

The serum concentration of TG in the CO group was significantly higher than in the CON group but was significantly lower than in the FO group (*p* < 0.05; Figure 5). The serum concentration of TG in MO had no significant difference with the CO, FO, and ASP groups (*p* > 0.05). Compared with the CON group, serum TC and LDL-C were significantly higher, but HDL-C was significantly lower in the CO, FO, MO, and ASP groups (*p* < 0.05). No significant difference was observed in TC, LDL-C, and HDL-C between the CO, FO, MO, and ASP groups (*p* > 0.05).

Compared with the CON group, the CO group had a significantly higher serum IL-6, IL-1β, IL-10, TNF-α, NF-κB, VCAM-1, and MCP-1 (*p* < 0.05; Figure 5). The MO and FO groups had a significantly lower serum IL-1β, NF-κB, and VCAM-1 than in the CO group (*p* < 0.05). No significant difference was observed in serum inflammatory factors between the MO and FO groups (*p* > 0.05).

### 3.3 Effect of mussel oil on inflammatory factors in the aorta

**Figure 6:**
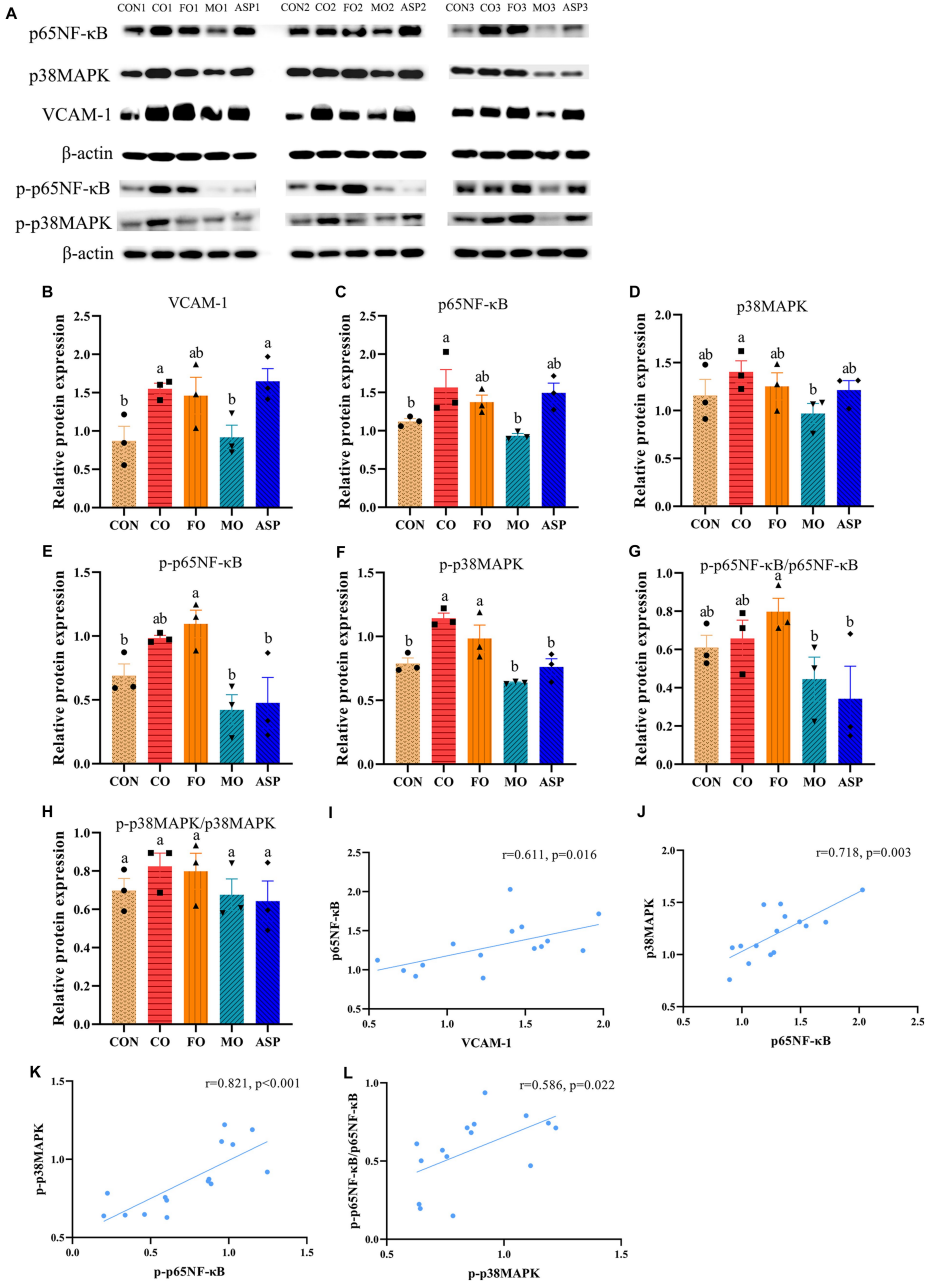
Effect of treatment oils on inflammatory factors in the aorta (*n* = 3 in each group). **(A)** Representative bands of Western blotting assay. **(B–F)** Quantitative results of VCAM-1, p65NF-κB, p38MAPK, p-p65NF-κB, and p-p38MAPK, respectively (mean ± SEM). **(G,H)** Ratios of p-p65NF-κB/p65NF-κB and p-p38MAPK/p38MAPK (mean ± SEM). **(I–L)** Significant correlations between inflammatory factors in the aorta (Spearman correlation). There was significance if groups did not share the same letter (*p* < 0.05). CON, health control; CO, corn oil; FO, fish oil; MO, mussel oil; ASP, aspirin.

The protein content of VCAM-1, p65NF-κB, and p-p38MAPK in the aorta was significantly higher in the CO group than in the CON group (*p* < 0.05; Figure 6). Compared with the CO group, the MO group but not the FO or ASP group had significantly lower VCAM-1 content in the aorta (*p* < 0.05), and this content in the MO group was comparable to the CON group (*p* > 0.05). The MO group but not the FO or ASP group had a significantly lower protein content of p65NF-κB and p38MAPK in the aorta than in the CO group (*p* < 0.05). The MO and ASP groups had a significantly lower protein level of p-p65NF-κB and the ratio of p-p65NF-κB/p65NF-κB than in the FO group and a lower protein level of p-p38MAPK than in the CO and FO groups (*p* < 0.05), and these contents in the MO and ASP groups were comparable to the CON group (*p* > 0.05). Aortic p65NF-κB was positively correlated with p38MAPK (*r* = 0.718, *p* = 0.003) and VCAM-1 (*r* = 0.611, *p* = 0.016); p-p65NF-κB was positively correlated with p-p38MAPK (*r* = 0.821, *p* < 0.001); the ratio of p-p65NF-κB/p65NF-κB was positively correlated with p-p38MAPK (*r* = 0.586, *p* = 0.022).

### 3.4 Fatty acid composition of erythrocyte membrane phospholipids

C18:2n-6, C20:4n-6, and total n-6 PUFA of erythrocyte membrane PL were significantly lower in the FO and MO groups than in the CON, CO, and ASP groups (*p* < 0.05; Supplementary Figure S1). C20:5n-3, C22:6n-3, and total n-3 PUFA and the ratio of n-3/n-6 PUFA were significantly higher in the FO and MO groups than in the CON, CO, and ASP groups (*p* < 0.05). No significant difference was observed in these PL PUFA contents between the FO and MO groups (*p* > 0.05).

### 3.5 Correlation between erythrocyte membrane phospholipid fatty acids and atherosclerosis-related parameters

**Figure 7:**
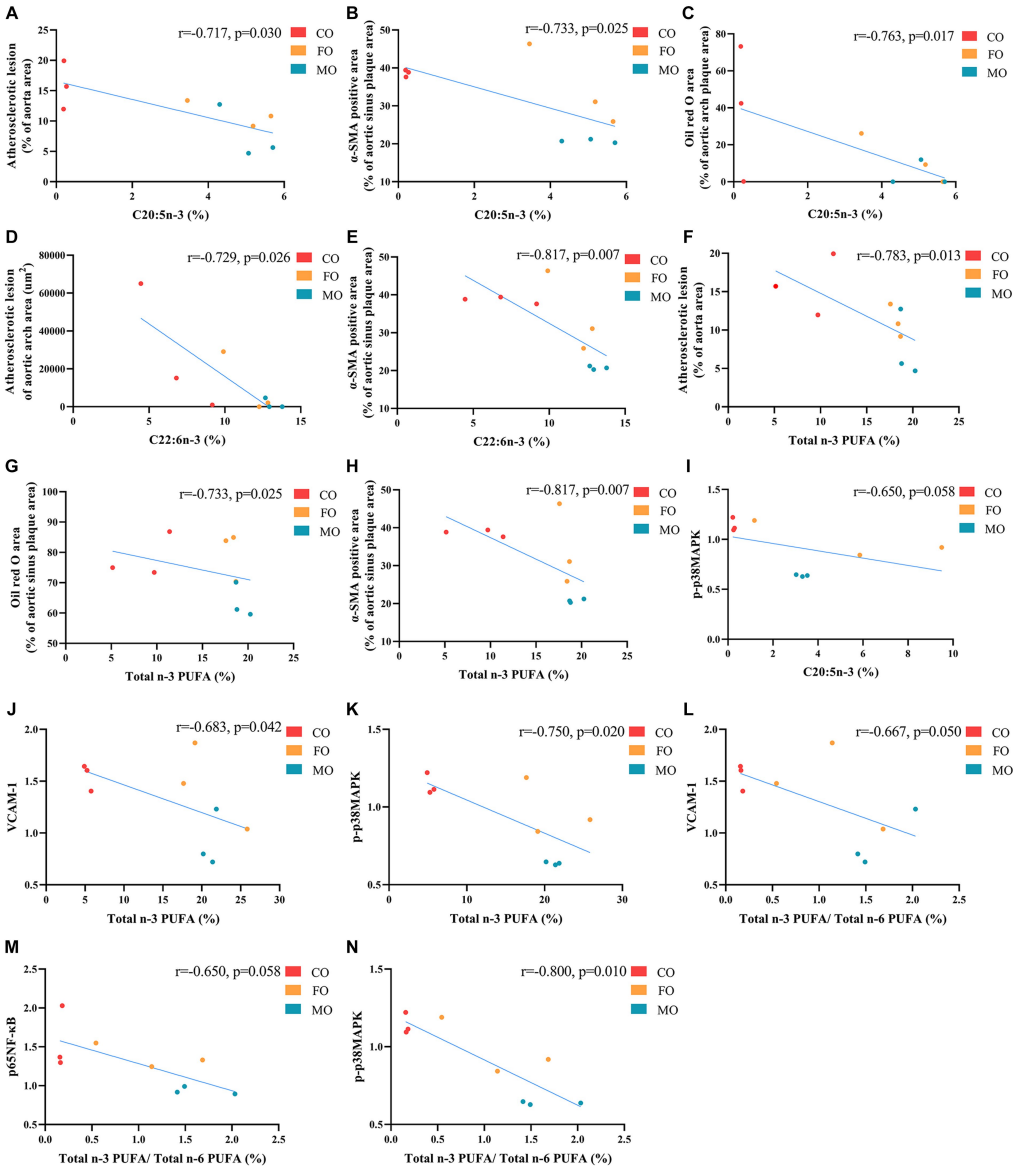
Correlation of n-3 polyunsaturated fatty acids between erythrocyte membrane phospholipids and atherosclerosis-related parameters (*n*  =  9). **(A-C, I)** The correlation between C20:5n-3 (%) and atherosclerotic lesion (% of aorta area), α-SMA positive area (% of aortic sinus plaque area), Oil red O area (% of aortic arch plaque area) and p-p38MAPK respectively. **(D-E)** The correlation between C22:6n-3 (%) and atherosclerotic lesion of aortic arch area and α-SMA positive area (% of aortic sinus plaque area) respectively. **(F,H,J,K)** The correlation between Total n-3 PUFA (%) and atherosclerotic lesion (% of aorta area), Oil red O area (% of aortic sinus plaque area), α-SMA positive area (% of aortic sinus plaque area), VCAM-1 and p-p38MAPK respectively. **(L-N)** The correlation between Total n-3 PUFA/ Total n-6 PUFA (%) and VCAM-1, p65NF-κB and p-p38MAPK. Spearman correlation analysis was used for data analysis. Mice in CO, FO and MO were included in correlation analysis.

Erythrocyte membrane PL C20:5n-3 and total n-3 PUFA were negatively correlated with atherosclerotic lesion area of the aorta (*r* = −0.717, *p* = 0.030; *r* = −0.783, *p* = 0.013) and SMC of the aortic sinus (indicated by α-SMA; r = −0.733, *p* = 0.025; *r* = −0.817, *p* = 0.007; Figure 7). PL C20:5n-3 was negatively correlated with lipid deposition (Oil red O staining) of the aortic arch (*r* = −0.763, *p* = 0.017), and PL total n-3 PUFA was negatively correlated with lipid deposition of the aortic sinus (*r* = −0.733, *p* = 0.025). PL C22:6n-3 was negatively correlated with atherosclerotic lesion area of the aortic arch (*r* = −0.729, *p* = 0.026) and SMC of the aortic sinus (*r* = −0.817, *p* = 0.007). p-p38MAPK was negatively correlated with PL C20:5n-3 (*r* = −0.650, *p* = 0.058), PL n-3 PUFA (*r* = −0.750, *p* = 0.020), and the ratio of PL n-3/n-6 PUFA (*r* = −0.800, *p* = 0.010). p65NF-κB was negatively correlated with the ratio of PL n-3/n-6 PUFA (*r* = −0.650, *p* = 0.058). VCAM-1 was negatively correlated with PL n-3 PUFA (*r* = −0.683, *p* = 0.042) and the ratio of PL n-3/n-6 PUFA (*r* = −0.667, *p* = 0.050).

Erythrocyte membrane PL C20:5n-3 was negatively correlated with serum IL-1β (*r* = −0.623, *p* = 0.006), NF-κB (*r* = −0.641, *p* = 0.004), and VCAM-1 (*r* = −0.536, *p* = 0.022; Supplementary Table S2). PL C22:6n-3 was positively correlated with serum TG (*r* = 0.569, *p* = 0.014). PL C22:6n-3 and total n-3 PUFA were negatively correlated with serum IL-1β (*r* = −0.663, *p* = 0.003; *r* = −0.657, *p* = 0.003), MCP-1 (*r* = −0.536, *p* = 0.022; *r* = −0.581, *p* = 0.011), NF-κB (*r* = −0.806, *p* < 0.001; *r* = −0.773, *p* < 0.001), TNF-α (*r* = −0.470, *p* = 0.049; *r* = −0.517, *p* = 0.028), and VCAM-1 (*r* = −0.692, *p* = 0.001; *r* = −0.672, *p* = 0.002). The ratio of PL n-3/n-6 PUFA was positively correlated with serum HDL-C (*r* = 0.523, *p* = 0.026) and negatively correlated with serum IL-1β (*r* = −0.599, *p* = 0.009), MCP-1 (*r* = −0.511, *p* = 0.030), NF-κB (*r* = −0.711, *p* = 0.001), and VCAM-1 (*r* = −0.550, *p* = 0.018).

### 3.6 The content of furan fatty acids and astaxanthin in treatment oils

The contents of 11D3 and 11D5 in FO were 3906.91 ng/mg and 1971.60 ng/mg. The content of astaxanthin was under detection limit in FO. The MO and CO used in the present study were the same as those used in our previous study (26). The contents of 11D3 and 11D5 in MO were 2828.70 ng/mg and 1582.10 ng/mg, the contents of 11D3 and 11D5 in CO were 210.92 ng/mg and 167.79 ng/mg, the content of astaxanthin was 191 mg/kg in MO and was under detection limit in CO, according to the previous study (26).

### 3.7 Serum furan fatty acids and its correlation with atherosclerosis-related parameters

**Figure 8:**
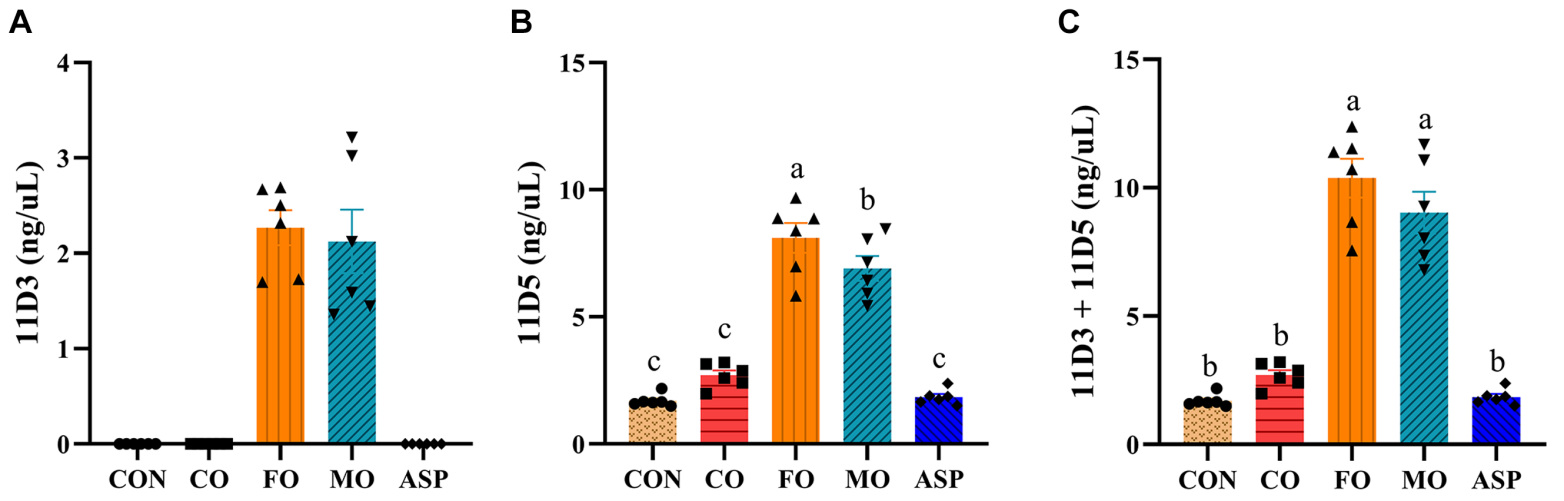
Effect of treatment oils on furan fatty acids in serum (*n*  =  6 in each group). The results of 11D3 **(A)**, 11D5 **(B)** and 11D3+11D5 **(C)**. Data were expressed as mean  ±  SEM. There was significance if groups did not share the same letter (*p * <  0.05). CON, health control; CO, corn oil; FO, fish oil; MO, mussel oil; ASP, aspirin.

The contents of 11D3 and 11D5 in serum are shown in Figure 8. No significant difference in 11D3 was found between the FO and MO groups (*p* > 0.05). The content of 11D3 in the serum of the CON, CO, and ASP groups did not reach the detection limit. The content of 11D5 in the MO group was significantly lower than that in the FO group (*p* < 0.05) but significantly higher than that in the CON, CO, and ASP groups (*p* < 0.05). Serum 11D5 was positively correlated with aortic p-p38MAPK/p38MAPK (*r* = 0.803, *p* = 0.016), aortic VACM-1 (*r* = 0.805, *p* = 0.016), and serum TG (*r* = 0.669, *p* = 0.003) after adjusting for erythrocyte membrane PL n-3 PUFA. The mass spectrum of 11D3-AMMP and 11D5-AMMP in serum is shown in Supplementary Figures S2, S3.

## 4 Discussion

In the present study, MO was more potent than FO in preventing atherosclerosis of ApoE^−/−^ mice, which provided a new strategy for nutritional prevention of atherogenesis. Commercial products of MO already existed, such as Lyprinol (PCSO-524), a lipid extract of the green-lipped mussel. Several clinical trials of MO have been conducted and found its beneficial effect on rheumatoid arthritis (14), osteoarthritis (28), asthma (29), and attention-deficit hyperactivity disorder (ADHD) (30). No obvious adverse effect of MO was reported during clinical trials (14, 28), indicating its safety. Future RCT can be conducted to clarify whether MO has an anti-atherosclerosis effect on humans and thus provide a basis for its application as a nutritional product for prevention and treatment of atherosclerosis.

The major fatty acids in both MO and FO are long-chain n-3 PUFA (mainly C20:5n-3 and C22:6n-3). The beneficial effect of n-3 PUFA on atherosclerosis has been reported in cohort studies (7, 8). A previous study showed that FO led to reduced aortic root lesions in ApoE^−/−^mice (31). In the present study, we observed that total n-3 PUFA content in erythrocyte membrane phospholipids was negatively correlated with the Oil Red O area of the aortic sinus (Figure 7G). However, no significant difference was observed in atherosclerotic area and Oil Red O area of the aortic sinus between the FO and CO groups. Possible reasons for this inconsistency are as follows. ApoE^−/−^ mice develop arterial lesions in a time-dependent manner (32). The age of mice in the present study for starting intervention was 7 weeks, but the age in the study by Wang et al. was 10 weeks (31). Difference in the age of mice can lead to variation in severity of atherosclerosis and may influence the treatment effect. In addition, during adaption, all mice were fed with AIN-93G diet for 1 week in the present study. As reported in our previous study (33), this diet contains 7% soybean oils (w/w) and 6% C18:3n-3 (% in total fatty acids). C18:3n-3 can be converted to C20:5n-3, C22:5n-3, and C22:6n-3 *in vivo* after desaturation and elongation. However, the fat of diet in the control group of the study by Wang et al. was provided by CO and did not contain C18:3n-3 (31). Therefore, the C18:3n-3 in the adaption diet may narrow the gap of n-3 PUFA content in mice tissue between the FO and CO groups and thus led to non-significant results. Conflicting results were also observed in clinical trials. An RCT found that pure C20:5n-3 supplementation (1.8 g/d) significantly decreased carotid intima-media thickness (IMT) of T2DM patients (34). However, another RCT found that the supplementation of fish oil (2.4 g/d, containing 35% C20:5n-3 and 20% C22:6n-3) had no effect on carotid IMT, plaque score, and plaque area in subjects with hypercholesterolaemia (35). Non-significant result was also observed in an RCT with fish oil treatment (3–6 g/d, containing 55% C20:5n-3 and C22:6n-3) (36). The anti-atherosclerosis effect of n-3 PUFA was supported by a recent meta-analysis of clinical trials with high dose of pure n-3 PUFA (≥1.8 g/d), but subgroup analysis indicated that a significant effect was only observed for pure C20:5n-3 but not mixture of C20:5n-3 and C22:6n-3 (37). These results indicated that type of n-3 PUFA, purity, and dosage may influence its anti-atherosclerosis effect. Therefore, for fish oil (a common source of n-3 PUFA), sources of fish and different preparation methods may also influence its beneficial effect on atherosclerosis. In the present study, lipid deposition of the aorta and aortic arch in the MO group was significantly lower than in the CO group; aortic atherosclerotic plaque area in the FO group was also slightly lower than in the CO group, although this difference was non-significant. The present study found that PL n-3 PUFA of erythrocyte membrane was negatively correlated with atherosclerotic plaque area of the aorta and aortic arch, lipid deposition of the aortic arch and sinus, and SMC content of the aortic sinus, indicating that n-3 PUFA is a bioactive compound which is responsible for the beneficial effect of MO on atherogenesis. However, n-3 PUFA cannot completely explain why MO was more potent than FO in preventing atherogenesis. In the present study, the content of total n-3 PUFA in MO and FO was comparable. Although MO had a slightly higher C22:6n-3 and a slightly lower C20:5n-3 than FO, there was no significant difference in PL C20:5n-3, C22:6n-3, and total n-3 PUFA in erythrocyte membrane between the MO and FO groups. Therefore, other bioactive components, except for n-3 PUFA in MO, may also have a strong anti-atherogenesis property.

In the present study, there was limited atherosclerotic lesion in the aortic arch. According to previous studies, ApoE^−/−^ mice aged 18 weeks (38) and ApoE^−/−^ mice fed a high-fat diet for 12 weeks also showed limited lipid deposition in the aortic arch (39). Aortic arch is a vulnerable site for atherosclerotic plaque formation in ApoE^−/−^ mice (40). However, one study observed that lipid deposition in the abdominal aorta was more than that in the aortic arch (20). Another study found that atherosclerotic plaque was higher in the aortic sinus than in the aortic arch (39). These were consistent with the present study.

The better anti-atherogenesis effect of MO than FO may be attributed to its good anti-inflammatory property. Our previous randomized controlled trial and animal study found that MO could improve arthritis by decreasing pro-inflammatory factors (such as TNF-α and PGE_2_) and increasing anti-inflammatory factors (such as IL-10) (14, 16). The anti-inflammatory effect of MO was also observed in mice with inflammatory bowel disease (41). In the present study, although the lowering effect of MO and FO on serum pro-inflammatory factors was comparable, only MO but not FO led to significantly lower aortic NF-κB, p-NF-κB, p38MAPK, and p-p38MAPK levels than CO. NF-κB and p38MAPK can upregulate the expression of pro-inflammatory factors, such as TNF-α, IL-6, and IL-1β (42–44). Both NF-κB and p38MAPK play a key role in atherosclerotic lesion formation (44, 45).

The oil composition of MO and FO differs in two aspects, which can help explain the better anti-atherogenesis effect of MO than FO. On one hand, in the present study, only MO but not FO contains a high level of astaxanthin. Overwhelming evidence suggests that astaxanthin may have a preventive effect on atherosclerotic cardiovascular disease through its potential to improve inflammation, lipid metabolism, and oxidative stress (46). Astaxanthin remarkably suppressed the expression of various inflammatory mediators, such as TNF-α, IL-1β, and IL-6 (47). It can also reduce the release of inflammatory cytokines in mice through the MAPK pathway and NF-κB pathway (48, 49). In addition, astaxanthin has a strong antioxidant ability, which has been shown to reduce cellular oxidative stress, DNA damage, and cell death through Nrf2-antioxidant response element pathway (50). On the other hand, the content of furan fatty acids in MO was lower than FO. Although several studies suggested potential benefits of furan fatty acids in inhibiting lipid peroxidation and reducing inflammation, including reducing risk factors associated with cardiovascular disease (15, 51), the contribution of furan fatty acids to human health remains uncertain. Some studies even reported its unfavorable effects. One study demonstrated that 11D3 exacerbated hepatic steatosis and acute kidney injury in diabetic mice and may also increase the risk of coronary heart disease in T2DM patients (52). Furan fatty acid 3-carboxy-4-methyl-5-propyl-2-furanpropanoic acid (CMPF), a metabolite of furan fatty acids and n-3 PUFA (53), has been reported to be enriched in the plasma of patients with chronic renal failure, gestational diabetes mellitus, and T2DM (54–56). However, an opposite result was observed in a recent study, which found that CMPF in T2DM patients was lower than in healthy people (57). CMPF also acts as a pro-oxidant leading to renal cellular damage (58). In the present study, serum 11D5 levels were significantly higher in the FO group than in the MO group, and 11D5 was found to be positively correlated with aortic p-p38MAPK/p38MAPK, VCAM-1, and serum TG. Therefore, we speculate that MO has better anti-atherosclerotic effects than FO, which may be due to the fact that MO contains more astaxanthin and less 11D5.

NF-κB is a transcription factor and plays an essential role in inflammation and immunity (59). It is necessary for cytokine-induced transcription of VCAM-1 (60), and p38MAPK can upregulate VCAM-1 expression at post-transcriptional level (61). p65 and p50 are two members of the NF-κB family (62). In the steady state, the dimers composed of p65 and p50 are retained within the cytoplasm by the IκB proteins. When the cell is stimulated, it leads to the ubiquitination of IκBs and subsequent degradation, inducing the phosphorylation of IκB protein and resulting in the release of p-p65NF-κB from cytoplasmic restraint. Released pp65NF- κB can drive target gene transcription and induce proinflammatory cytokine expression (63). In the present study, steady-state NF-κB was measured in serum, and activated p-p65NF-κB was measured in the aorta. The content of p-NF-κB in the aorta was significantly lower in the MO group than in the FO group, while there was no significant difference in serum NF-κB in the present study. Some previous studies have also shown inconsistent results for NF-κB and p-NF-κB (64, 65), indicating that there is no necessary connection between the levels of NF-κB and p-NF-κB. In the present study, aortic protein content of VCAM-1 was positively correlated with aortic protein contents of NF-κB. VCAM-1 can recruit monocytes to activate vascular endothelium (1). The recruited monocytes can differentiate into macrophages and further transform into foam cells by the uptake of modified LDL (such as oxidized LDL), leading to the formation of lipid plaque (45). In the present study, MO but not FO significantly decreased the aortic protein level of VCAM-1, and the MO group had a slightly lower level of macrophages in the aortic sinus than in the FO group. This can help explain why MO was more potent than FO in the prevention of atherogenesis. In addition, oxidized LDL can enhance the uptake of macrophages and lead to cholesterol ester accumulation and foam cell formation (66). One previous study found that p38MAPK is necessary for oxidized LDL-induced lipid uptake of macrophages and foam cell formation (67). In the present study, both p38MAPK and p-p38MAPK in the aorta were decreased by MO but not FO, implying that MO may also inhibit the influx of cholesterol into macrophages and foam cell formation. However, this has not been verified in the present study and future study is needed to clarify this point.

SMC plays a dual role in atherosclerosis progression (45): On one hand, abnormal proliferation, migration, and cell growth of SMC are driving factors of atherosclerosis during the development of this disease, leading to thickened intima layer of the blood vessel wall and thus reduced blood flow; on the other hand, after atherosclerotic plaque was formed, SMC can protect against plaque rupture by forming a protective layer around lipid cores. In the present study, the SMC content in the MO and ASP groups was significantly lower than in the CO and FO groups. The present study aimed to evaluate the preventive effect of MO on atherosclerosis (intervention was started before serious atherosclerotic plaque was formed) rather than the treatment effect after serious plaque was formed. Therefore, the lowering effect of MO on SMC content reflected a protective role of MO against the development of atherosclerosis. The p38MAPK can accelerate the proliferation of pulmonary artery SMC (68) and is involved in airway SMC migration (69). The important role of p38MAPK in proliferation and migration of vascular SMC was observed *in vitro* study (70). In addition, NF-κB is also involved in migration of SMC (71, 72). Therefore, inhibiting proliferation and migration of SMC by downregulating the p38MAPK/NF-κB signaling pathway is another possible mechanism for the anti-atherogenesis effect of MO.

The present study had several limitations. First, serum astaxanthin was not detected in the present study because the serum was used for detecting other parameters. Future studies are necessary to clarify the relationship between astaxanthin and atherosclerosis-related parameters in biological samples. Second, the results have not been verified in human studies, and thus, caution should be exercised when extending the results to humans. Third, in the present study, the results about the underlying mechanism are preliminary, and future study is warranted to further dig into the potential mechanism, such as the influence of MO on influx and efflux of cholesterol in macrophages. Fourth, the sample size of the present study was small. We will verify the results with larger sample size in future studies.

In conclusion, the MO group had significantly smaller atherosclerotic plaque area, lower lipid deposition, lower contents of SMC, and slightly lower contents of macrophage at the aortic sinus than in the FO group. Compared with the CO group, MO but not FO had significantly lower lipid deposition in the aortic arch, smaller atherosclerotic plaque area, and lower inflammatory factors in the aorta. Therefore, MO is more potent than FO in preventing atherosclerosis. The possible mechanism may be by downregulating p38MAPK/NF-κB signaling pathway, decreasing VCAM-1 and macrophages, and inhibiting proliferation and migration of SMC. Considering that the total n-3 PUFA in MO and FO was comparable, the anti-atherosclerotic effect of MO was better than FO because MO contains more astaxanthin and less 11D5.

## Data availability statement

The original contributions presented in the study are included in the article/Supplementary material, further inquiries can be directed to the corresponding author.

## Ethics statement

The animal study was approved by the Ethics Committee of Medical College of Qingdao University (QDU-AEC-2022369). All animal experimental procedures were performed in accordance with the Guidelines for Care and Use of Laboratory Animals of Qingdao University. The study was conducted in accordance with the local legislation and institutional requirements.

## Author contributions

KL: Conceptualization, Funding acquisition, Methodology, Resources, Supervision, Validation, Writing – original draft, Writing – review & editing. XS: Data curation, Formal analysis, Investigation, Methodology, Software, Visualization, Writing – review & editing. HL: Project administration, Validation, Writing – review & editing. XK: Project administration, Validation, Writing – review & editing. SL: Investigation, Project administration, Validation, Writing – review & editing. RL: Funding acquisition, Project administration, Writing – review & editing. DL: Conceptualization, Funding acquisition, Resources, Supervision, Writing – review & editing.
