# Supplementary material for: Mussel oil is superior to fish oil in preventing atherosclerosis of ApoE−/− mice
Source: Front Nutr. 2024 Feb 12;11:1326421. doi: 10.3389/fnut.2024.1326421 (PMC10894946; doi:10.3389/fnut.2024.1326421)
Supplement: Supplementary file 1 [file Data_Sheet_1.docx]

Supplementary Material

**Table S1.** Fatty acids composition of treatment oils (% in total fatty acids).

| Fatty acids | CO | FO | MO |
| --- | --- | --- | --- |
| C8:0 | 0.10 | 0.01 | ND |
| C12:0 | 0.07 | 0.01 | ND |
| C14:0 | 0.08 | 0.48 | 3.36 |
| C15:0 | ND | 0.2 | 0.44 |
| C16:0 | 10.83 | 13.42 | 19.13 |
| C17:0 | ND | 0.37 | 0.66 |
| C18:0 | 3.65 | 1.08 | 2.04 |
| C20:0 | ND | 0.58 | ND |
| C23:0 | ND | 0.43 | 1.23 |
| Total SFA | 14.73 | 16.58 | 26.85 |
| C14:1n-5 | ND | 0.04 | 0.11 |
| C16:1n-7 | ND | 4.19 | 11.89 |
| C17:1n-7 | ND | ND | 0.44 |
| C18:1n-9 | 26.27 | 23.81 | 3.20 |
| C20:1n-9 | ND | 3.99 | 3.26 |
| C22:1n-9 | ND | 1.34 | ND |
| Total MUFA | 26.27 | 33.37 | 18.91 |
| C18:3n-3 | 5.54 | 1.64 | 2.38 |
| C20:3n-3 | ND | 0.08 | 0.17 |
| C20:5n-3 | ND | 25.27 | 20.66 |
| C22:5n-3 | ND | 0.49 | 1.07 |
| C22:6n-3 | ND | 19.34 | 26.72 |
| Total n-3 PUFA | 5.54 | 46.82 | 51.01 |
| C18:2n-6 | 53.47 | 0.65 | 1.14 |
| C18:3n-6 | ND | 0.17 | ND |
| C20:2n-6 | ND | 0.78 | 0.66 |
| C20:3n-6 | ND | 0.64 | ND |
| C20:4n-6 | ND | 0.99 | 1.43 |
| Total n-6 PUFA | 53.47 | 3.23 | 3.23 |

SFA, saturated fatty acid; MUFA, monounsaturated fatty acid; PUFA, polyunsaturated fatty acid; FO, fish oil; MO, mussel oil; CO, corn oil; ND, not detected or negligible.

**Table S2.** Correlation between PL n-3 PUFA in erythrocyte membrane and serum lipids and inflammatory factors.

| Parameters | C20:5n-3 | | C22:6n-3 | | Total n-3 PUFA | | Total n-3 PUFA/ Total n-6 PUFA | |
| --- | --- | --- | --- | --- | --- | --- | --- | --- |
|  | r | p | r | p | r | p | r | p |
| TG (mmol/L) | 0.350 | 0.155 | 0.569 | 0.014 | 0.298 | 0.229 | 0.455 | 0.058 |
| TC (mmol/L) | 0.150 | 0.553 | 0.327 | 0.185 | 0.040 | 0.874 | 0.197 | 0.433 |
| HDL-C (mmol/L) | 0.150 | 0.553 | 0.154 | 0.542 | 0.383 | 0.117 | 0.523 | 0.026 |
| LDL-C (mmol/L) | 0.265 | 0.287 | 0.288 | 0.247 | 0.292 | 0.240 | 0.406 | 0.095 |
| IL-1β (pg/mL) | -0.623 | 0.006 | -0.663 | 0.003 | -0.657 | 0.003 | -0.599 | 0.009 |
| IL-6 (pg/mL) | -0.141 | 0.576 | -0.385 | 0.115 | -0.269 | 0.280 | -0.251 | 0.316 |
| IL-10 (pg/mL) | -0.046 | 0.855 | -0.220 | 0.381 | -0.263 | 0.291 | -0.179 | 0.478 |
| MCP-1 (pg/mL) | -0.437 | 0.070 | -0.536 | 0.022 | -0.581 | 0.011 | -0.511 | 0.030 |
| NF-κB (pg/mL) | -0.641 | 0.004 | -0.806 | <0.001 | -0.773 | <0.001 | -0.711 | 0.001 |
| TNF-α (pg/mL) | -0.410 | 0.091 | -0.470 | 0.049 | -0.517 | 0.028 | -0.432 | 0.073 |
| VCAM-1 (ng/mL) | -0.536 | 0.022 | -0.692 | 0.001 | -0.672 | 0.002 | -0.550 | 0.018 |

Mice in CO, FO and MO groups were included in correlation analysis.


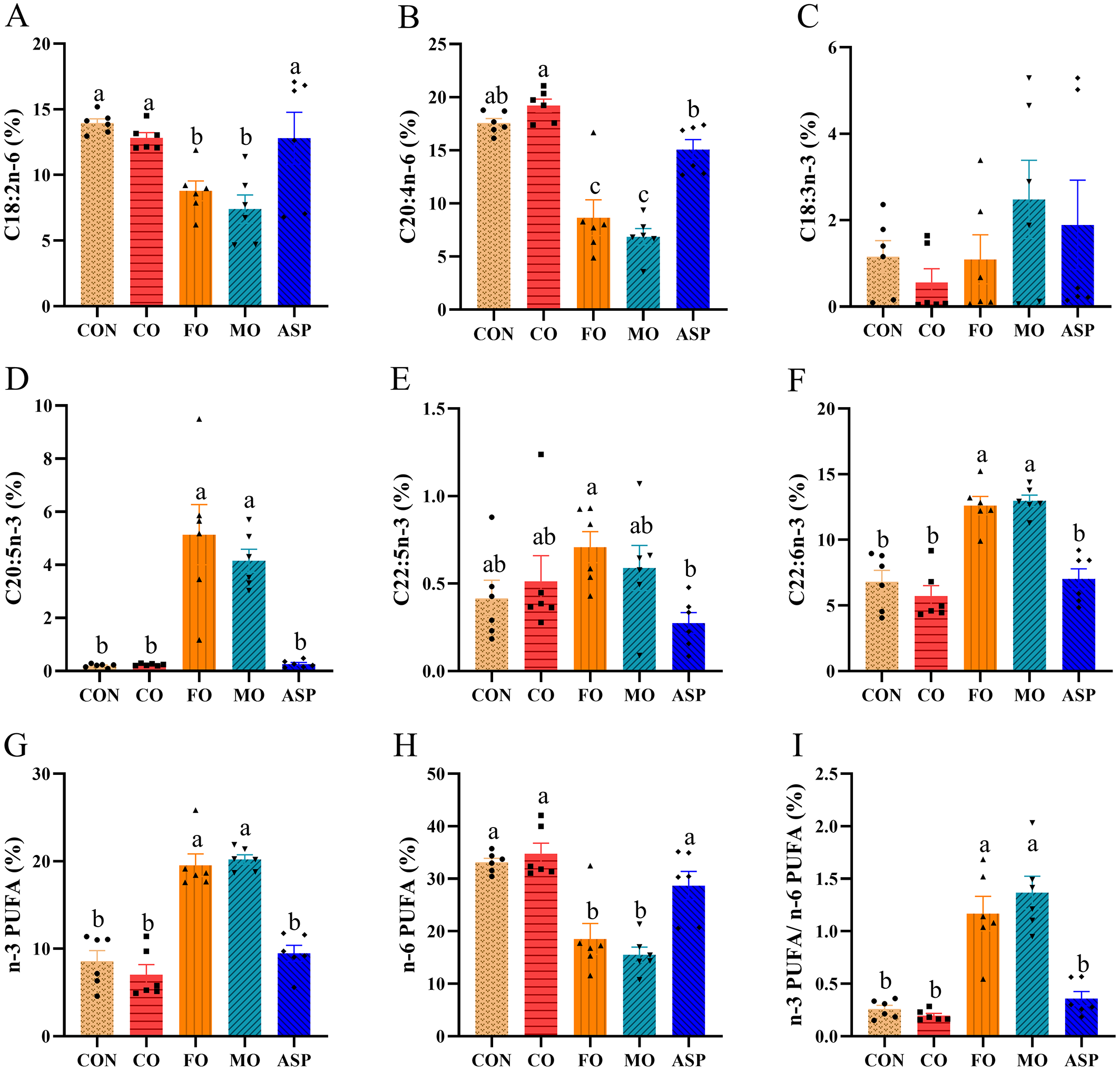


**Figure S1.** Polyunsaturated fatty acids in erythrocyte membrane phospholipids. There was significance if groups did not share the same letter (p < 0.05). PUFA, polyunsaturated fatty acids; CON, health control; CO, corn oil; FO, fish oil; MO, mussel oil; ASP, aspirin.

**
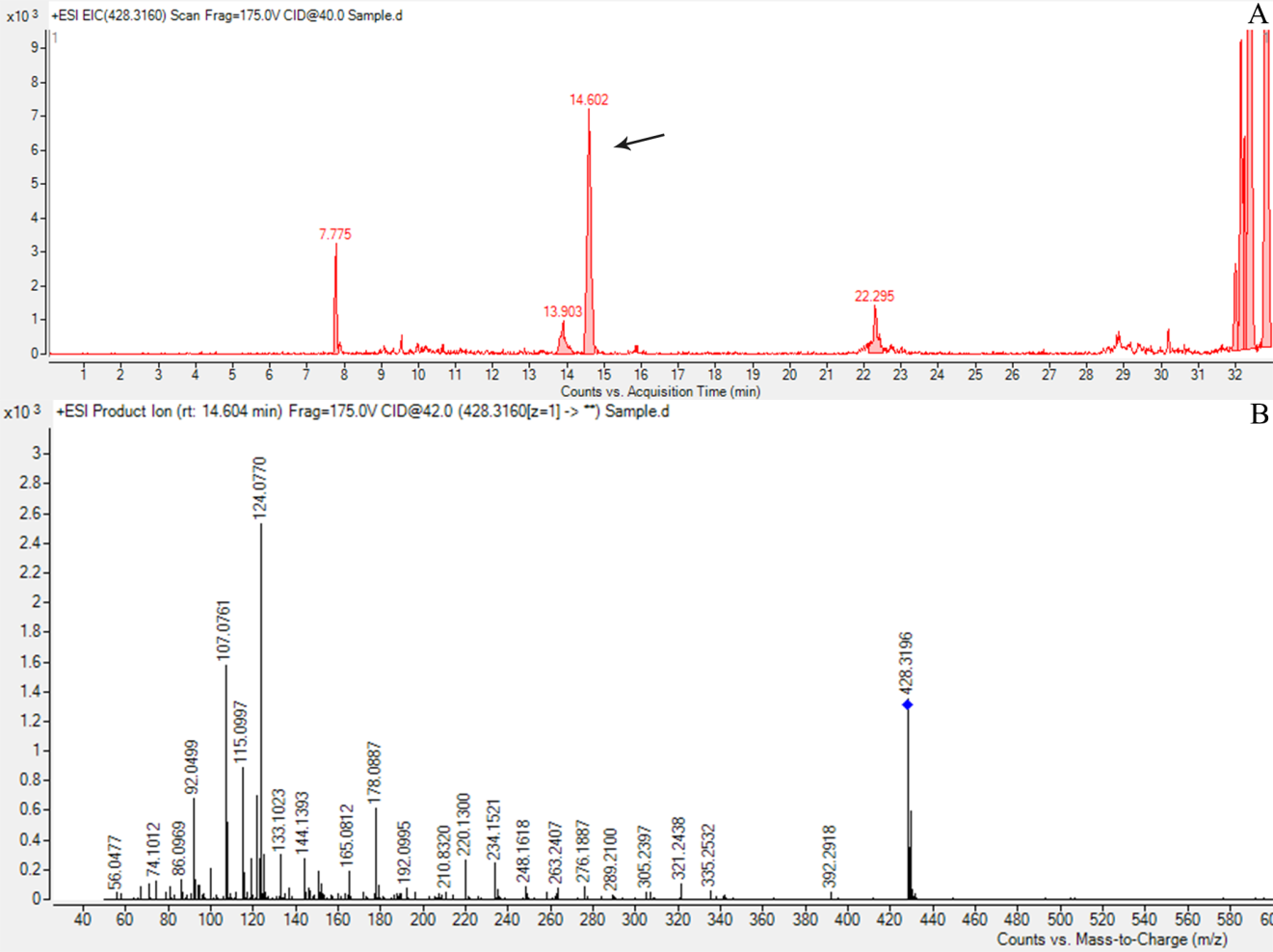
**

**Figure S2.** The MS and MS/MS spectrum of 11D3-AMMP in serum.

**
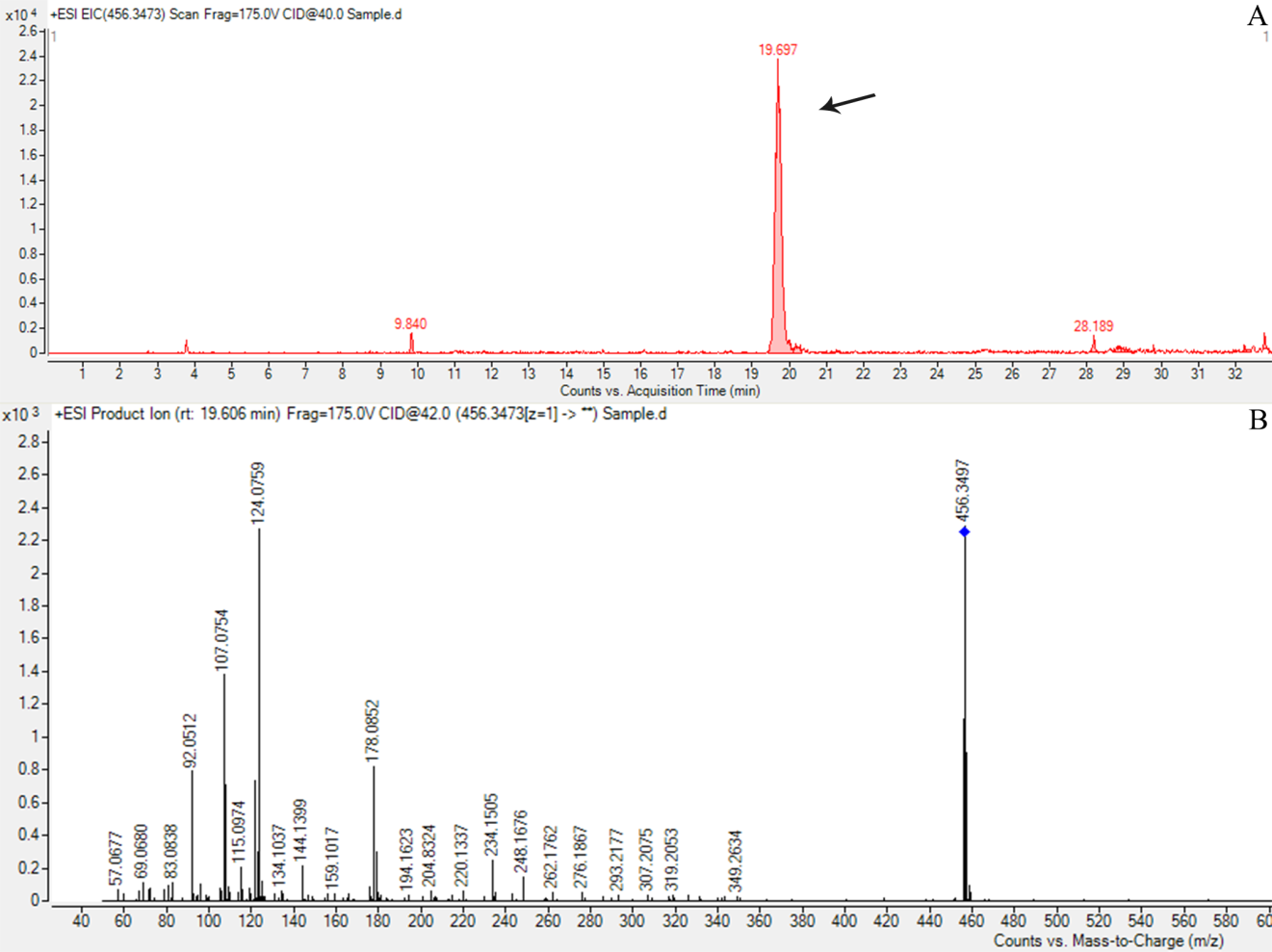
**

**Figure S3.** The MS and MS/MS spectrum of 11D5-AMMP in serum.
